# Draft Genome Sequence of “*Candidatus* Mycoplasma haemobos,” a Hemotropic Mycoplasma Identified in Cattle in Mexico

**DOI:** 10.1128/genomeA.00656-16

**Published:** 2016-07-07

**Authors:** Fernando Martínez-Ocampo, Sergio D. Rodríguez-Camarillo, Itzel Amaro-Estrada, Rosa Estela Quiroz-Castañeda

**Affiliations:** Unidad de Anaplasmosis, Centro Nacional de Investigación Disciplinaria en Parasitología Veterinaria, INIFAP, Jiutepec, Morelos, Mexico

## Abstract

We present here the draft genome sequence of the first “*Candidatus* Mycoplasma haemobos” strain found in cattle in Mexico. This hemotropic mycoplasma causes acute and chronic disease in animals. This genome is a starting point for studying the role of this mycoplasma in coinfections and synergistic mechanisms associated with the disease.

## GENOME ANNOUNCEMENT

Hemotropic mycoplasmas (hemoplasmas) are the causative agent of infectious anemia in a variety of mammalian species (1). Bovine hemoplasmas are cell wall-less bacteria that can be found attached to surface-bound or free erythrocytes in the plasma of cattle (2). “*Candidatus* Mycoplasma haemobos” (syn. “*Candidatus* Mycoplasma haemobovis) may cause anemia and depression to various degrees, edema, reproductive problems, and various other clinical signs in cattle (3). These bacteria have been reported in cattle from several countries around the world and may act in a synergistic manner when coinfections are present (4–8). In this study, we report the draft genome sequence of “*Candidatus* Mycoplasma haemobos” INIFAP01, an uncultivated hemoplasma found in blood of sick cattle from Chihuahua, Mexico. Genomic DNA was obtained using the UltraClean DNA BloodSpin kit (Mo Bio Laboratories). Five micrograms of genomic DNA were sequenced in the HiSeq 2000 system (Illumina). We obtained a random data set of 45,165,900 paired-end reads of 300 bases long. Quality-based trimming was performed with a DynamicTrim (SolexaQA++) Perl script, and genome assembly was accomplished *de novo* using the SPAdes (version 3.1.1) program. The draft genome has 18 contigs, with a calculated 935,638-bp total length, *N*_50_ contig size of 256,799 bp, G+C content of 30.46% and ~48× coverage.

We identified the 16S rRNA gene of “*Ca.* Mycoplasma haemobos” INIFAP01 using the RNAmmer 1.2 server (http://www.cbs.dtu.dk/services/RNAmmer/) and compared it with 30 16S rRNA gene sequences of the genus *Mycoplasma* and two 16S rRNA genes of the genus *Ureaplasma* (outgroup). All sequences were aligned with the MUSCLE server (http://phylogeny.lirmm.fr/phylo_cgi/one_task.cgi?task_type=muscle), and a phylogenetic analysis was performed in the MEGA (version 6.1) program with a neighbor-joining algorithm using 1,000 replicates for bootstrapping. Phylogenetic analysis reveals that “*Ca.* Mycoplasma haemobos” INIFAP01 is closely related to the *Mycoplasma haemobos* species. The 16S rRNA gene of “*Ca.* Mycoplasma haemobos” INIFAP01 is 1,460 bp in length.

The 18 contigs were analyzed with the RAST server (http://rast.nmpdr.org/), identifying 1,216 open reading frames (ORFs) and 1,184 coding sequences (CDSs). This study was approved by the Centro Nacional de Investigación Disciplinaria en Parasitología Veterinaria (CENID-PAVET) branch of the Instituto Nacional de Investigaciones Forestales Agrícolas y Pecuarias (INIFAP) Animal Experimentation and Ethics Committee and conducted considering ethic and methodological aspects, in agreement with the Mexican regulations related to use, housing, and transport of experimental animals, no. NOM-062-ZOO-1999.

### Nucleotide sequence accession numbers.

This whole-genome shotgun project has been deposited at DDBJ/ENA/GenBank under the accession no. LWUJ00000000. The version described in this paper is version LWUJ01000000.
